# Accidental portal vein catheterization during pleural drainage catheter insertion: a case report

**DOI:** 10.1186/s13256-023-04291-4

**Published:** 2023-12-20

**Authors:** Sohaib Zoghoul, Israa Al‑Hashimi, Qayed Aldebyani, Rahil Kassamali, Ahmed Omar, Ali Barah

**Affiliations:** Department of Radiology, Hamad Medical Corporation, Doha, Qatar

**Keywords:** Iatrogenic injuries, Portal vein, Pleural drainage catheter, PDC insertion, Complications, Hemorrhage, Interventional radiology, Management, Case report

## Abstract

**Background:**

Iatrogenic portal vein (PV) injuries following pleural drainage catheter (PDC) insertion are rare but life-threatening. This case report emphasizes the importance of prompt recognition and effective interventional radiology (IR) management.

**Case presentation:**

A 38-year-old Asian male, admitted for a non-ST-segment elevation myocardial infarction, suffered a critical PV injury during PDC insertion, leading to rapid clinical deterioration. The IR team conducted a portogram, retrieved the catheter, and successfully executed an embolization procedure. The patient's recovery, confirmed through imaging and improving liver function tests, enabled discharge with follow-up instructions.

**Conclusions:**

This case highlights the clinical significance of promptly recognizing and effectively managing iatrogenic PV injuries during PDC insertion, with the pivotal role of IR. Collaboration between IR and surgical teams is crucial for optimizing patient outcomes.

## Introduction

Iatrogenic PV injury resulting from the insertion of a PDC on the right side is a rare but potentially severe complication. The PV is responsible for transporting nutrient-rich blood from the gastrointestinal tract to the liver. Damage to this pathway can have significant consequences, including mortality. PDC insertion is a common procedure used for diagnosis and treatment, but complications can arise when performed near the liver and its associated blood vessels. Unintentional insertion of the catheter within the liver can lead to iatrogenic injury to the PV. The immediate risk of such an injury can result in uncontrolled bleeding, requiring urgent intervention. Moreover, interrupted blood flow in the PV system induced by the catheter can affect liver function, leading to complications like hepatic ischemia, portal hypertension, and the potential development of varices. Detecting a PV injury requires a high level of suspicion and the use of appropriate imaging techniques such as computed tomography (CT) or angiography to visualize the location, extent, and associated complications. Swift identification and accurate characterization are crucial for determining the most appropriate management strategies. Treating accidental PV injuries following PDC insertion requires a multidisciplinary approach involving IR and surgery. The primary goals of treatment are to achieve hemostasis, restore PV flow, and minimize long-term complications. The choice of treatment method depends on factors such as the extent and location of the injury, the patient's condition, and the expertise of the medical team. Collaborative decision-making between interventional radiologists and surgeons is vital in determining the most appropriate strategy and achieving successful outcomes for patients with iatrogenic PV injuries. The purpose of this article is to present a rare case of an accidental portal vein catheterization during PDC insertion.

## Case presentation

A 38-year-old Asian male patient, with a 20-year history of chronic smoking at a rate of 2 packs per day, a past history of alcohol consumption that ceased 6 years ago, and untreated hypertension, presented with sudden onset epigastric pain lasting 20 min the night before admission. The following morning at 9 am, the patient experienced blurring of vision, mild dizziness, and exertional chest pain. The electrocardiogram (ECG) revealed ST-segment depression and T-wave changes, while serial troponin levels rose from 37 to 39 and then to 42. An echocardiogram showed a significant drop in the ejection fraction (EF) to 40%. Given these findings, the patient was admitted as a case of non-ST-segment elevation myocardial infarction (NSTEMI) and underwent coronary angiography (CAG), which revealed triple-vessel disease (3VD) suitable for coronary artery bypass grafting (CABG). There were no reported allergies, and socially, the patient is employed as a driver, working an 8-h light job with sufficient rest breaks.

**Fig. 1:**
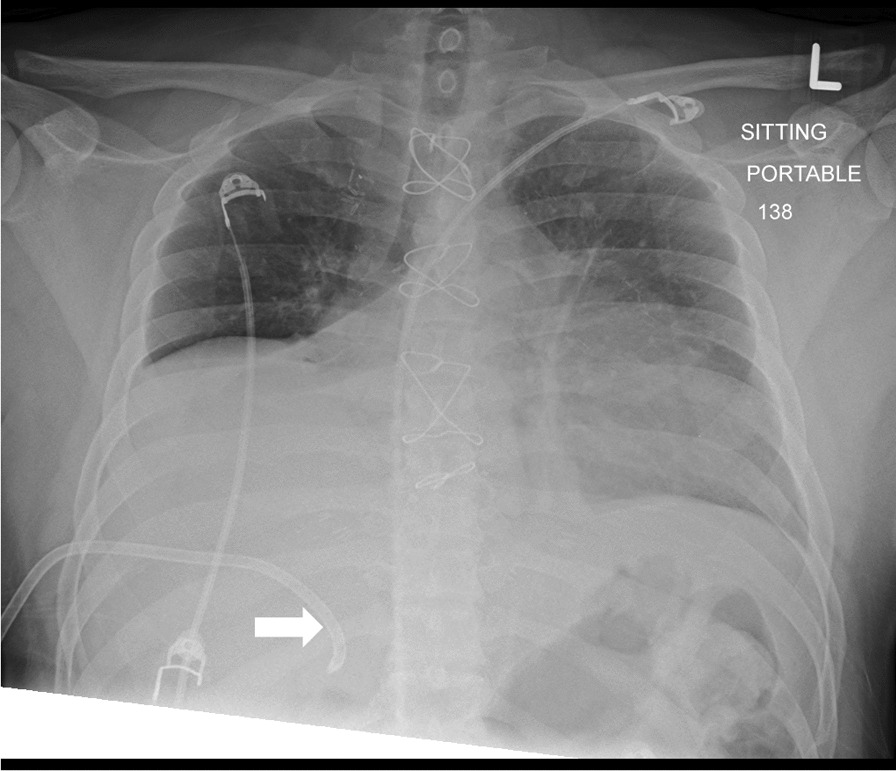
The post-pleural drainage catheter insertion portable sitting chest X-ray demonstrates the presence of a right-sided pleural drainage catheter (white arrow) passing through the 7th intercostal space, with its tip positioned over the liver shadow. Unfortunately, the primary physician misinterpreted this as a right-sided large pleural effusion

During the hospital course, a chest X-ray (Fig. 1) revealed a large right pleural effusion, leading to the insertion of a right-sided pleural drainage catheter (PDC) by an experienced cardiothoracic physician. The blind 12F PDC insertion involved procedural sedation with local lidocaine 2%, utilizing aseptic measures with the patient in a semi-right lateral position. Initial attempts at the 5th right intercostal space yielded no fluid, prompting a shift to the 6th intercostal space posteriorly, where dark blood was successfully drained using a pigtail needle. Despite the procedure, post-examination showed no improvement in air entry, and the entire process took approximately 25 min. Shortly after insertion, the patient's condition deteriorated, manifesting as dizziness, hypotension, and tachycardia. The right PDC rapidly drained approximately 1300 ml of old blood within one hour. Vital signs included a temperature of 36.9°C, heart rate of 83 bpm, respiratory rate of 19 br/min, systolic blood pressure of 106 mmHg, diastolic blood pressure of 65 mmHg, and oxygen saturation of 99%. Neurological status remained intact, with a Glasgow Coma Scale (GCS) of 15/15, equal and reacting pupils. Motor examination showed the ability to move all limbs. Renal function indicated adequate urine output, and the gastrointestinal examination revealed a soft abdomen. Hematology abnormalities included a high white blood cell count (WBC), low red blood cell count (RBC), low hemoglobin (Hgb), and low hematocrit (Hct). Chemistry results showed normal renal and electrolyte parameters, except for a high ALT level. Prothrombin time was elevated, and medications included aspirin, bisoprolol, clopidogrel, furosemide, lactulose, lidocaine, pantoprazole, and paracetamol, with additional PRN medications administered as needed.

**Fig. 2:**
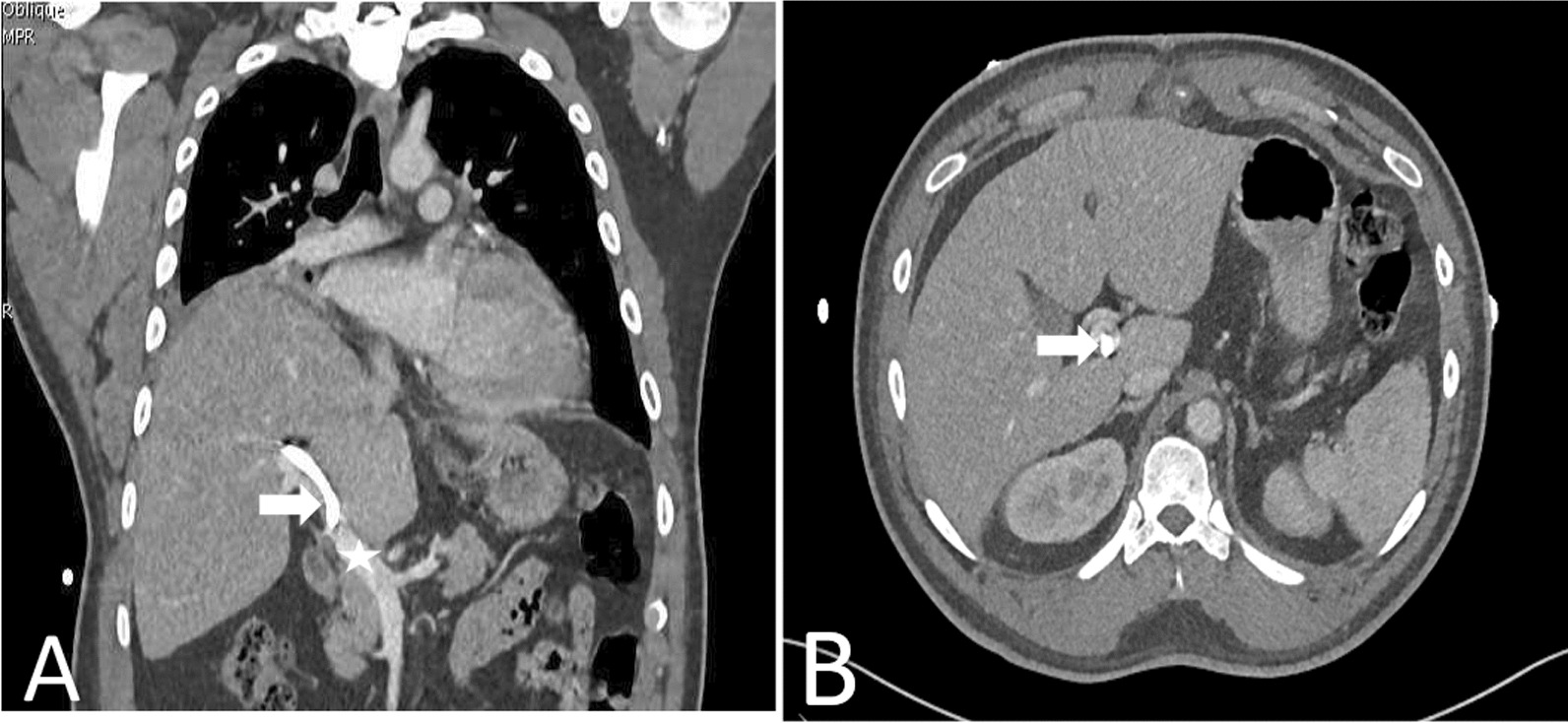
Coronal (**A**) and axial (**B**) CT images of the chest and abdomen with contrast enhancement reveal the presence of a right-sided pleural drainage catheter (white arrow) traversing through the right 7th intercostal space and penetrating the liver parenchyma, with the catheter tip positioned within the main portal vein (Star)

Subsequent CT thorax imaging (Fig. 2) revealed the unexpected presence of the PDC traversing through the right hepatic lobe and terminating within the distal main PV. The imaging also identified right lower lung lobe collapse, occluded bronchus, and an elevated right hemidiaphragm. However, there was no evidence of active bleeding or significant intraperitoneal free fluid, and small bilateral pleural effusions were noted.

**Fig. 3:**
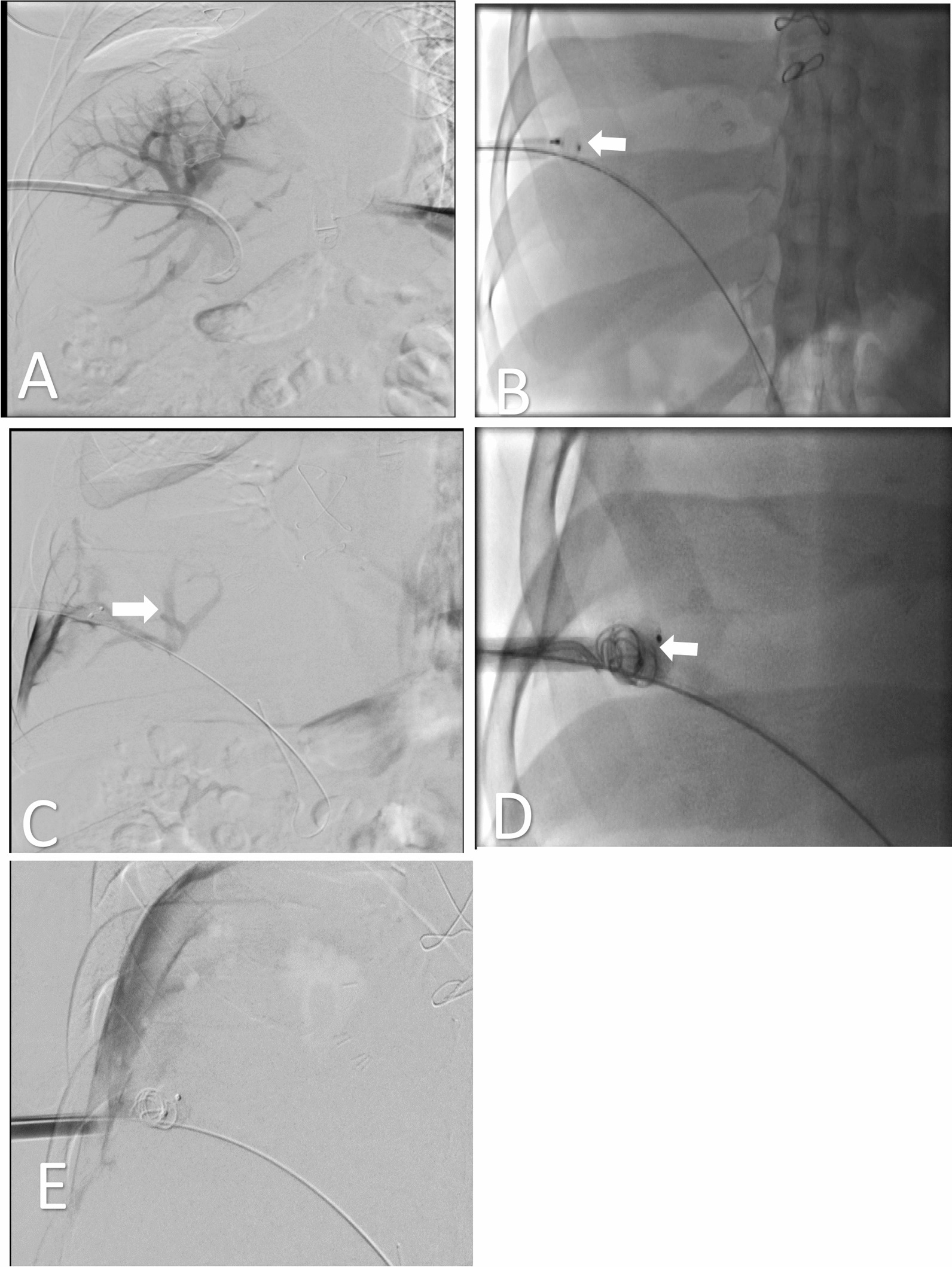
Illustrative images of interventional fluoroscopy and Digital Subtraction Angiography (DSA) showcasing the portal vein. **A** Injection of contrast via the misplaced right-sided pleural catheter allows visualization of the main portal vein and its branches, confirming the catheter tip's location (White arrow) in the main portal vein (Star). **B** Following catheter removal and the introduction of a wire, a 10 × 7 mm vascular plug (White arrow) has been successfully deployed. **C** Digital Subtraction Angiography after vascular plug deployment reveals residual opacification in the portal vein branches (White arrow). **D** Two coils (White arrow) measuring 10 × 8 mm and 8 × 5 mm have been placed. **E** Final Digital Subtraction Angiography, following vascular plug and coil deployment, demonstrates complete absence of opacification in the main portal vein and its branches

Given the critical nature of the patient's condition, admission to the surgical intensive care unit (SICU) was warranted. The interventional radiology (IR) team was consulted and advised to perform a portogram prior to any further management. A portogram through the PDC confirmed PV patency and the absence of peritoneal extravasation (Fig. 3). Catheter retrieval under fluoroscopy guidance was performed over a stiff guidewire, followed by placing a 12F introducer sheath for intrahepatic track embolization. A 10 mm Amplatzer vascular plug (AVP) type 2 was placed at the tract, with additional embolization using 10 mm and 8 mm coils. Gel foam slurry was injected distal to the coils and proximal to AVP for further embolization of the tract.

Following the embolization procedure, the patient was closely monitored in the intensive care unit. Serial Doppler ultrasound examinations confirmed the absence of peritoneal hematoma, reflecting the successful occlusion of the intrahepatic parenchymal track with restoration of normal PV flow. Laboratory parameters, including liver function tests, gradually improved over the following days, indicating recovery. Hemoglobin improved from 6 to 8.9 gm/dL, and ALT decreased from 250 to 63 U/L. Despite these anomalies, the patient's vital signs and overall physical condition appeared stable, with normal blood pressure, heart rate, and oxygen saturation. The patient was discharged with appropriate instructions for follow-up and continued monitoring.

In the follow-up, the patient maintained a medication regimen comprising aspirin (enteric coated) at 100 mg, bisoprolol at 2.5 mg, clopidogrel at 75 mg, pantoprazole at 40 mg, and rosuvastatin at 20 mg, all administered orally. Notably, the patient expressed a willingness to quit smoking, reported no instances of chest pain, and successfully resumed work. The review of systems indicated unremarkable relevant symptoms. The physical examination revealed vital signs within normal limits. The overall appearance was well, and the wound was observed to be dry and clean.

## Discussion

This case presents a distinctive interventional radiology perspective involving accidental portal vein catheterization during pleural catheter drainage (PCD) insertion. Unlike reported cases, this incident led to the catheter traversing the right hepatic lobe into the distal main portal vein, a rare complication. The swift deterioration post-insertion necessitated urgent intervention, emphasizing the crucial role of interventional radiology in promptly recognizing and managing unforeseen complications during routine procedures. The subsequent portogram, catheter retrieval, and intrahepatic track embolization underscore the complexity and uniqueness of this case, contributing novel insights to the existing interventional radiology literature.

PCD is commonly performed for various diagnostic and therapeutic purposes, such as draining pleural effusions, ascites, or abscesses. However, complications can arise, and can be categorized in two types: catheter-related complications including pleural infections, cellulitis, empyema, tunnel infections, and catheter blockage or dislodgment, and procedure-related complications including pneumothorax, subcutaneous emphysema, bleeding, trauma to intrathoracic and intraabdominal structures. One such potential rare complication is vascular iatrogenic injury of the portal vein (PV), especially when the procedure is performed near the liver and its associated vasculature.

PV plays a crucial role in transporting blood enriched with essential nutrients from the gastrointestinal tract to the liver, making any compromise to this vessel life-threatening [1]. The mechanism of injury usually involves penetrating trauma with overall mortality ranging between 45 to 71% [1, 2]. Despite advancements in overall survival rates for traumatic incidents, outcomes related to PV injuries have not significantly improved in recent decades [3].

The consequences of PV injury can manifest in multiple ways. The immediate risk includes hemorrhage and hemodynamic instability due to the high flow within the PV. Additionally, impaired blood flow in the PV system can lead to severe implications for liver function, causing hepatic ischemia, portal hypertension, and the potential development of varices. The diagnosis of a PV injury requires a high level of suspicion and appropriate imaging modalities such as CT or angiography to identify the location, extent, and associated complications accurately [4].

The management of accidental PV injury after right sided PCD insertion requires a multidisciplinary approach involving interventional radiology (IR) and surgical teams. The primary goal is to achieve hemostasis, restore PV flow, and minimize the risk of long-term complications. Treatment options may include surgical repair, cover stent placement, PV embolization, TIPPS or conservative management for less severe injuries.

Conservative management can be considered for less severe PV injuries due to low profile needle or catheter iatrogenic trauma. Close observation, supportive care, and monitoring of the patient's clinical status, along with appropriate imaging follow-up, can be employed to ensure that the injury does not progress or cause significant clinical complications. In cases induced by higher profile materials, percutaneous track embolization is an additional secure way to minimize the risk of peritoneal bleeding. Various surgical treatment methods are also available. Primary repair is feasible for early identification and small caliber PV injuries, while venography is utilized for larger or complex PV injuries [5, 6]. Adjunctive techniques, such as reinforcement with vascular patches or grafts, may enhance the durability of the repair in challenging cases. Extensive PV injuries may require PV resection and reconstruction using various techniques, such as end-to-end anastomosis, interposition grafts, or venous conduit.

IR treatment methods offer alternative options as well. PV embolization, a minimally invasive procedure performed by interventional radiologists [7], can be carried out through a percutaneous transhepatic approach, either ipsilateral (through the lobe of the liver requiring PV embolization) or contralateral [8]. In rare instances, a trans splenic approach may be safely performed when the ipsilateral route presents challenges [7]. Regardless of the approach chosen for gaining access into the PV, the steps of the procedure remain consistent. The technical success rate of PV embolization is nearly 100%, with acceptable guidelines recommending low rates of complications [8, 9]. Trans jugular Intrahepatic Portosystemic Shunt (TIPS) is another well-established IR procedure that diverts blood flow away from the injured PV segment [10]. However, TIPS carries a moderate risk of complications, particularly bleeding, both during and after the procedure [11, 12]. Covered stent placement is an advanced IR technique used for managing larger or extensive iatrogenic PV injuries [13]. This technique involves deploying a covered stent across the injured segment to create a durable and patent conduit [14]. Percutaneous covered stent placement has been successfully utilized to treat post-traumatic bleeding cases [15].

The selection of the most appropriate treatment method depends on various factors, including the extent and location of the PV injury, the patient's clinical status, and the expertise available. A multidisciplinary approach involving interventional radiologists and surgeons is essential for determining the optimal treatment strategy and achieving successful outcomes for patients with iatrogenic PV injuries [16].

### Conclusion

IR plays a pivotal role in the management of iatrogenic PV injuries following PDC insertion. This case report underscores the significance of prompt recognition, expert procedural planning, and technical proficiency in achieving successful outcomes. Collaborative efforts between IR and surgical teams are essential to ensure the best possible care for patients with iatrogenic PV injuries. Further research and knowledge sharing in this field will contribute to the refinement of treatment algorithms and improved patient outcomes.

## Data Availability

Not applicable.

## Abbreviations

PV: Portal vein
IR: Interventional radiology
PDC: Pleural drainage catheter
CT: Computed tomography
